# The double-edged sword of regulatory T cells in MASLD: from inflammation control to fibrosis promotion

**DOI:** 10.1080/19768354.2026.2630463

**Published:** 2026-02-16

**Authors:** So Eun Lee, Min Gi Jo, Sang Won Park, Hye Jung Kim, Seung Pil Yun, Jong-Won Kim

**Affiliations:** a Department of Pharmacology, Institute of Medical Science, College of Medicine, Gyeongsang National University, Jinju, Republic of Korea; b Department of Convergence Medical Science, College of Medicine, Gyeongsang National University, Jinju, Republic of Korea; c Department of Pathology, College of Medicine, Kyung Hee University, Seoul, Republic of Korea

**Keywords:** Liver, MASLD, MASH, liver fibrosis, regulatory T cell

## Abstract

Metabolic dysfunction-associated steatotic liver disease (MASLD) and its advanced inflammatory subtype, metabolic dysfunction-associated steatohepatitis (MASH), are becoming increasingly prevalent liver disorders driven by complex interactions between metabolic stress, immune dysregulation, and fibrotic remodeling. Regulatory T cells (Tregs), traditionally recognized for their immunosuppressive functions, have emerged as key modulators of hepatic inflammation, fibrosis, and systemic metabolic balance. While Tregs can suppress pro-inflammatory immune responses and mitigate liver injury, accumulating evidence highlights their paradoxical roles in liver fibrosis, including the promotion of hepatic stellate cell activation and profibrotic remodeling. This review critically examines the dual and context-dependent roles of Tregs in MASLD and MASH, emphasizing implications for therapeutic intervention. Additionally, we discuss current preclinical strategies aimed at modulating Treg abundance and function, highlighting the challenges and opportunities associated with developing stage-specific therapeutic approaches that consider not only local hepatic effects but also potential systemic metabolic consequences.

## 1. Introduction

Metabolic dysfunction-associated steatotic liver disease (MASLD) has recently replaced the term nonalcoholic fatty liver disease to reflect the underlying metabolic risk factors and pathophysiological basis of the disease better (Hsu and Loomba 2024). MASLD represents the hepatic manifestation of systemic metabolic dysfunction, encompassing a spectrum from benign hepatic steatosis to a more aggressive subtype, known as metabolic dysfunction-associated steatohepatitis (MASH). MASH is characterized by hepatocyte injury, lobular inflammation, and progressive fibrosis (Loomba et al. 2021). With a global prevalence exceeding 25%, MASLD poses an escalating public health burden and is projected to become the leading cause of cirrhosis, liver failure, and hepatocellular carcinoma in the coming decades (Cotter and Rinella 2020).

Although MASLD has traditionally been regarded as a consequence of metabolic overload and lipotoxic stress, growing evidence has highlighted the critical contribution of immune dysregulation to its pathogenesis. The liver functions as a unique immunological organ that sustains immune tolerance despite continuous exposure to gut-derived microbial products, dietary nutrients, and xenobiotics delivered via the portal circulation. In MASLD, this finely tuned immunological equilibrium is perturbed, resulting in chronic low-grade inflammation and inappropriate activation of both innate and adaptive immune responses (Shrestha et al. 2025; Sutti and Albano 2020).

Among the key regulatory components of the immune system, regulatory T cells (Tregs) have emerged as pivotal mediators in maintaining hepatic immune tolerance and modulating inflammatory responses. Tregs are a specialized subset of CD4^+^ T cells characterized by the expression of the transcription factor forkhead box P3 (FOXP3) and high levels of CD25, also known as the interleukin-2 (IL-2) receptor alpha chain. These cells exert immunosuppressive effects through multiple mechanisms, including cytokine secretion, metabolic disruption of effector T cells, and modulation of antigen-presenting cells (APCs) (Bilate and Lafaille 2012; Josefowicz et al. 2012; Wing et al. 2019). Emerging evidence indicates that Tregs are not only critical regulators of hepatic immune homeostasis but are also highly responsive to metabolic cues within the liver microenvironment. Their abundance, phenotypic characteristics, and suppressive function are modulated by a range of local factors, including the cytokine milieu, lipid-derived metabolites, bile acids, and gut microbiota-associated molecules, many of which are altered in MASLD (Furusawa et al. 2013; Song et al. 2020; Zhou et al. 2022).

This review aims to provide an overview of the pathophysiological mechanisms underlying MASLD and MASH, with particular emphasis on the functions of Tregs. Furthermore, we highlight the context-dependent roles of Tregs and explore their potential as therapeutic targets in MASLD and MASH.

## 2. Pathologic overview of MASLD and MASH

From a histopathological standpoint, MASLD is defined by the accumulation of macrovesicular fat within ≥5% of hepatocytes, with the initial changes typically localized to the pericentral (zone 3) region of the hepatic lobule. In MASH, a progressive form of MASLD, additional histologic features emerge, including lobular inflammation, hepatocyte ballooning, and varying degrees of fibrosis, most commonly perisinusoidal in distribution (Nakamura et al. 2025; Rho et al. 2025; Zhao et al. 2025). The earliest pathological event involves excessive retention of neutral lipids in hepatocytes, arising from increased free fatty acid (FA) uptake, enhanced de novo lipogenesis, impaired β-oxidation, and reduced very-low-density lipoprotein export (Puri et al. 2007). Although simple steatosis is often clinically silent, progression to MASH reflects a key transition mediated by lipotoxic injury, sterile and metabolic inflammation, and progressive fibrotic remodeling (Meyer et al. 2024).

Lipotoxicity, a hallmark of disease progression, results from the accumulation of hepatotoxic lipid species such as diacylglycerols, ceramides, and free cholesterol. These metabolites trigger cellular stress pathways, including mitochondrial dysfunction, endoplasmic reticulum stress, and oxidative injury, which collectively impair hepatocyte viability and promote programmed cell death via apoptosis, necroptosis, and ferroptosis (Afonso et al. 2024; Malhi and Gores 2008; Qi et al. 2020). Damaged hepatocytes release damage-associated molecular patterns (DAMPs), which activate pattern recognition receptors on Kupffer cells (KCs) and dendritic cells (DCs), initiating a robust innate immune response (Garcia-Martinez et al. 2016; Kim et al. 2018; Roh et al. 2018).

The innate immune system, including KCs, monocyte-derived macrophages, and dendritic cells, plays a central role in initiating hepatic inflammation by recognizing DAMPs and pathogen-associated molecular patterns translocated from the gut microbiota through a compromised intestinal barrier (Park et al. 2021). These activated immune cells produce pro-inflammatory cytokines such as tumor necrosis factor- α (TNF-α), IL-1β, and IL-6, as well as chemokines that recruit additional immune effectors, amplifying hepatocellular damage and sustaining inflammation.

Adaptive immune cells, including CD4^+^ and CD8^+^ T cells, and B cells, have been increasingly implicated in MASLD progression. In particular, proinflammatory subsets such as Th1, Th17, and cytotoxic CD8^+^ T cells are enriched in the livers of patients with MASH and contribute to hepatocellular injury through cytokine-mediated inflammation and direct cytotoxicity (Hammerich et al. 2014; Wang et al. 2021a; Wolf et al. 2014).

Concomitant with immune activation, hepatic stellate cells (HSCs), the primary fibrogenic cell type in the liver, are activated by inflammatory cytokines, reactive oxygen species, and apoptotic bodies from dying hepatocytes (Che et al. 2023; Kim et al. 2025). Upon activation, HSCs transdifferentiate into myofibroblast-like cells expressing α-smooth muscle actin and produce excessive extracellular matrix (ECM) components, including collagen types I and III (Kim and Kim 2024). Persistent ECM deposition disrupts the normal sinusoidal architecture, leading to capillarization, parenchymal distortion, and ultimately cirrhosis in advanced cases. The degree of liver fibrosis remains the most robust histological predictor of liver-related morbidity and mortality in patients with MASLD (Dulai et al. 2017).

These findings demonstrate that the progression of MASLD and MASH is governed by multiple interrelated factors, including metabolic disturbances, immune dysfunction, and fibrotic changes. Chronic inflammation plays a central role not only in initiating liver injury but also in perpetuating disease progression. Since Tregs are key modulators of immune tolerance and inflammation, and are increasingly recognized for their potential to counterbalance pro-inflammatory immune responses in the liver, a deeper understanding of their role and regulation in the immunopathogenesis of MASLD and MASH may offer valuable insights for therapeutic intervention.

## 3. Tregs: origin, function, and mechanisms

Tregs primarily develop in the thymus, where they are derived from CD4^+^CD8^+^ double-positive thymocytes. Most thymic Treg cells belong to the CD4^+^ lineage, comprising approximately 5% of mature CD4^+^ single-positive thymocytes (Sakaguchi 2005). Thymus-derived regulatory T cells (tTregs) can be functionally categorized into naïve-like central Tregs (cTregs), also referred to as resting Tregs, and activated effector Tregs (eTregs), also known as effector memory Tregs (Koizumi and Ishikawa 2019). Upon maturation, tTregs exit the thymus as cTregs, which display a CD62L^high^CD44^low^ or CCR7^high^CD44^low^ phenotype, and circulate through secondary lymphoid organs (Lucca and Dominguez-Villar 2020). In response to antigenic stimulation, these cTregs differentiate into eTregs, which are characterized by the downregulation of CD62L and C–C chemokine receptor type (CCR) 7 and the upregulation of various chemokine receptors, such as CCR4, CCR6, and CCR10, as well as adhesion molecules, including killer cell lectin-like receptor subfamily G member 1 and CD103. These phenotypic changes facilitate migration and retention within non-lymphoid peripheral tissues (Dias et al. 2017; Koizumi et al. 2018; Levine et al. 2014).

CD25, initially used to identify suppressive CD4^+^ T cells, is constitutively expressed by most endogenous CD4^+^ Tregs (Sakaguchi et al. 1995). Despite comprising only 5–10% of peripheral CD4^+^ T cells, Tregs are indispensable for immune tolerance and homeostasis, largely because of their immunosuppressive role in the adaptive immune response that ensures self-tolerance (Sakaguchi 2005). The development, stability, and suppressive functions of Tregs are tightly regulated by specific transcription factors. Among these, FOXP3 is a lineage-defining transcription factor essential for the regulation of immune responses under both physiological and pathological conditions (Workman et al. 2009). Mutations in the *FOXP3* gene impair the development and function of Tregs, resulting in immune dysregulation, autoimmunity, and the onset of immunodysregulation polyendocrinopathy enteropathy X-linked (IPEX) syndrome, a rare but severe autoimmune disorder in humans (Ochs et al. 2005). The scurfy mouse, a well-established model for IPEX, lacks functional Tregs due to a mutation in *Foxp3* and consequently develops fatal systemic autoimmune disease and premature death, despite Tregs comprising only a small proportion of the total immune cell population (Hadaschik et al. 2015). These findings underscore that CD4^+^FOXP3^+^ Tregs are indispensable for maintaining immune homeostasis, suppressing autoimmune responses, and limiting immunopathology-induced tissue damage.

A minor subset of CD8^+^FOXP3^+^ Treg cells has also been identified in both mice and humans, and emerging evidence suggests that these cells may also originate in the thymus, although their developmental pathways, phenotypic characteristics, and functional roles remain less well defined compared to those of CD4^+^FOXP3^+^ Tregs (hereafter referred to as Tregs) (Kim et al. 2010).

**Figure 1.**
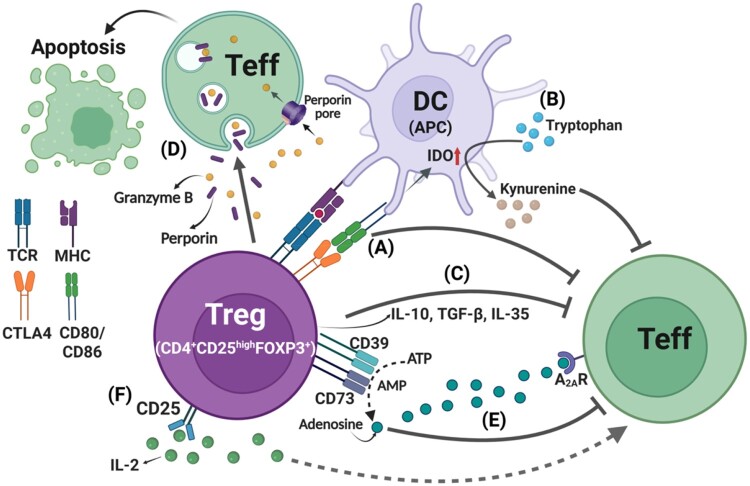
Mechanisms of immunosuppression mediated by Tregs. Tregs suppress immune responses via multiple mechanisms and maintain tissue homeostasis. (A) CTLA4-mediated suppression: Treg-expressed CTLA-4 inhibits DC co-stimulation by reducing CD80/CD86 availability, thereby suppressing Teff activation. (B) IDO induction by DC: CTLA-4 engagement with CD80/CD86 on DCs induces expression of IDO, which degrades tryptophan into kynurenine as immunoregulatory metabolites, suppressing Teff. (C) Immunosuppressive cytokine secretion: Tregs produce IL-10, IL-35, and TGF-β, which reduce inflammatory responses and inhibit Teff activation. (D) Granzyme B/perforin-mediated apoptosis: Tregs induce Teff apoptosis via granzyme B/perforin pathways and endocytotic uptake. (E) ATP-adenosine conversion: CD39 hydrolyzes extracellular ATP to AMP, and subsequently CD73 converts AMP to adenosine, which inhibits Teff responses via A_2A_R. (F) IL-2 deprivation mechanism via CD25^high^ Tregs: High expression of CD25^high^ on Tregs restricts IL-2 availability, limiting Teff expansion. A_2A_R; A_2A_ receptor, CTLA4; Cytotoxic T lymphocyte-associated antigen 4, DC; dendritic cell, IDO; Indoleamine 2,3-dioxygenase, Teff; effector T cell.

The immunosuppressive function of Tregs operates through several well-established mechanisms (Figure 1). (A) Cytotoxic T lymphocyte-associated antigen 4 (CTLA4) on Tregs that directly binds to the co-stimulatory molecules CD80 and CD86 on APCs (DCs), thereby suppressing APC function and preventing activation of effector T (Teff) cells (Gan et al. 2022). (B) Tregs can modulate APCs to upregulate indoleamine 2,3-dioxygenase (IDO), an immunoregulatory enzyme that catalyzes tryptophan degradation into proapoptotic metabolites. This process suppresses Teff responses via a pathway involving CTLA4-mediated interactions with CD80/CD86 on APCs. (C) Tregs secrete immunosuppressive cytokines such as IL-10, IL-35, and transforming growth factor-beta (TGF-β), which inhibit the activation and function of both Teff cells and APCs (Dikiy and Rudensky 2023). (D) Tregs induce Teff apoptosis by releasing granzyme B/perforin pathways and endocytotic uptake. (E) By expressing CD39 and CD73, Tregs convert extracellular ATP to adenosine, which binds to adenosine receptors on Teff cells and suppresses their activation (Deaglio et al. 2007; Ohta and Sitkovsky 2014). (F) Tregs competitively consume IL-2 via high levels of CD25, thereby limiting IL-2 availability for survival and proliferation of Teff cells (Caridade et al. 2013).

Overall, Tregs are involved in immunosuppressive functions, and tissue-resident eTregs play crucial roles in maintaining local immune homeostasis and promoting tissue repair (Koizumi and Ishikawa 2019). The role of Tregs has been emphasized not only in immunosuppressive functions but also in their protective or pathogenic roles in hepatic immune homeostasis.

## 4. Tregs in the microenvironment of MASLD and MASH

The roles of Tregs in MASLD and MASH remain unclear, with emerging evidence suggesting that their hepatic accumulation and functional relevance may vary depending on the disease model employed (Sawada et al. 2023). Although no distinct liver-specific Treg phenotype has been conclusively identified, multiple studies have reported that the core immunosuppressive capacity of Tregs remains largely preserved during the progression of MASLD and MASH (Ma et al. 2016).

One of the key pathogenic features of MASLD is hepatic lipid accumulation, which disrupts immune homeostasis and promotes chronic inflammation (Kim and Hyun 2024; Syed-Abdul 2023). Moreover, hepatic lipid accumulation is frequently accompanied by increased oxidative stress, a hallmark of disease progression from simple steatosis to steatohepatitis (Chen et al. 2020; Wang et al. 2024b). Elevated oxidative stress enhances Treg apoptosis, contributing to an overall decline in Treg abundance. Under non-inflammatory conditions, KCs, liver-resident macrophages, exhibit an M2-like phenotype, secrete anti-inflammatory cytokines, such as IL-10, and promote tissue repair and Treg differentiation (Breous et al. 2009; Dey et al. 2014). However, during chronic inflammation, the microenvironment required to maintain these tissue-resident macrophages is disrupted. As a result, KCs are progressively depleted and replaced by pro-inflammatory monocyte-derived macrophages, which produce cytokines such as IL-1β, TNF-α, IL-6, and osteopontin (Remmerie et al. 2020). This inflammatory milieu destabilizes *Foxp3* expression in Tregs and impairs their suppressive functions (Campbell and Koch 2011; Komatsu et al. 2014). In addition, elevated leptin levels have been shown to reduce the frequency of Tregs in both individuals with obesity and mouse models (Francisco et al. 2018). Tregs consistently exhibit an increased susceptibility to apoptosis in MASH (Sutti and Albano 2020).

Impaired suppression of hepatic inflammation may further promote progression from simple steatosis to steatohepatitis (Ma et al. 2007). Since Tregs are immunoregulatory cells capable of suppressing pro-inflammatory cytokine production by effector T cells, such as Th1 and Th17 cells (Workman et al. 2009), a reduction in Treg numbers under sustained disease conditions leads to an increased Th17/Treg imbalance and aberrant activation of pro-inflammatory T cell subsets, ultimately resulting in hepatocyte injury and exacerbation of liver inflammation (Komatsu et al. 2014; Zhang et al. 2021). Moreover, chronic inflammatory cues can destabilize *FOXP3* expression, downregulate *CD25*, and induce the phenotypic conversion of Tregs into Teffs, including Th1 and Th17, further amplifying the inflammatory response (Kleinewietfeld and Hafler 2013). This process of functional plasticity further impairs the immunoregulatory capacity of Tregs, potentially accelerating the development and/or progression of hepatic diseases (Komatsu et al. 2014; Wang et al. 2024a).

In addition, a growing body of evidence suggests that metabolic cues within the liver microenvironment significantly influence the fate and function of Tregs. Activated Tregs derive energy from multiple sources, including glucose, amino acids, FAs, and vitamins. In vitro studies have shown that lipid biosynthesis, aerobic glycolysis, and FA oxidation (FAO) contribute to ATP generation in Tregs (Wang et al. 2020). *FOXP3* expression in Tregs is associated with increased mitochondrial mass and elevated production of reactive oxygen species (ROS). Oxidative phosphorylation (OXPHOS) and ROS play critical roles in Treg signaling and homeostasis (Kempkes et al. 2019). Unlike other T cell subsets, Tregs depend heavily on FAO and OXPHOS for differentiation and function, whereas their proliferation relies on both glycolysis and FAO (Wang et al. 2017). Lactate serves as a substrate for the tricarboxylic acid cycle and contributes to the metabolic reprogramming in Tregs by enhancing mitochondrial OXPHOS (Zhou et al. 2024). This metabolic shift strengthens the suppressive capacity of Tregs, suggesting that elevated lactate levels may directly influence their function and support immune regulation (Zhou et al. 2024).

All-trans retinoic acid (ATRA), a vitamin A metabolite, contributes to the growth, differentiation, and functional regulation of Tregs (Liu et al. 2015). In the liver, HSCs produce ATRA, which enhances *Foxp3^+^* expression in CD4^+^ T cells and supports the development, stability, and activity of Tregs, particularly under inflammatory conditions (Dunham et al. 2013).

Given the connection between the gut and the liver through the portal vein, dietary components and microbial metabolites significantly influence Treg function in the liver. Growing evidence highlights the substantial impact of the gut microbiota and their metabolites on modulating immune responses (Zeng and Chi 2015). Indeed, most short-chain fatty acids (SCFAs) are generated through the fermentation of undigested dietary fiber in the presence of gut microbiota, which play essential roles in regulating immune responses. SCFAs regulate cellular metabolism by signaling via metabolite-sensing G protein-coupled receptors (GPCRs). Tregs express GPR43, a GPCR that recognizes SCFAs, thereby promoting Treg differentiation and enhancing their ability to maintain intestinal homeostasis. In particular, butyrate, an SCFA produced by gut bacteria, enhances histone acetylation at the *Foxp3* promoter by inhibiting histone deacetylase activity, thereby supporting the stable expression of *Foxp3*.

Taken together, the immunometabolic microenvironment in MASLD and MASH not only reduces the abundance of hepatic Tregs but also reprograms their phenotype and function. The dynamic and complex interplay of inflammatory and metabolic cues within the liver further shapes Treg behavior, modulating their stability and regulatory capacity. This disruption of Treg homeostasis contributes to the pathogenesis of chronic liver disease, reinforcing the relevance of Tregs as biomarkers and potential therapeutic targets.

## 5. Protective roles of Tregs in MASLD and MASH

The depletion of Tregs exacerbates hepatic inflammation in MASLD (Ma et al. 2007; Roh et al. 2018), suggesting that Tregs are crucial for regulating immune responses. As outlined in the previous section, Tregs are often reduced in number or functionally impaired in MASLD/MASH livers, contributing to uncontrolled Teff activation and enhancing Th1- and Th17-mediated hepatic pathology. The hepatic microenvironment in MASLD favors Th17 differentiation by upregulating retinoic acid-related orphan receptor gamma t (RORγt) expression while concurrently destabilizing FOXP3 in Tregs, leading to their phenotypic conversion into pro-inflammatory subsets (Zhang et al. 2021). Th17 cells and their signature cytokine IL-17 have been implicated in promoting hepatic steatosis and amplifying proinflammatory responses, thereby facilitating the transition from simple steatosis to steatohepatitis (Tang et al. 2011). Furthermore, a monokine induced by interferon-gamma (also known as CXCL9) has been shown to promote Th17 differentiation and expansion via JNK signaling, while concurrently reducing the relative abundance of Tregs, thereby disrupting the Th17/Treg balance, which is essential for immune regulation (Li et al. 2021). Given these findings, therapeutic strategies aimed at restoring or modulating the Th17/Treg balance warrant further investigation as potential interventions to prevent or reverse disease progression to MASH.

In addition to IL-17, the inflammatory hepatic microenvironment characterized by elevated levels of TNF-α compromises the survival of Tregs and promotes their apoptosis during hepatocyte injury and the progression of MASH. In a previous study, TLR7 signaling induced DCs to produce type I IFN and KCs to produce TNF-α, which in turn triggered the suppression and apoptosis of Tregs, ultimately impairing their protective role in MASH (Roh et al. 2018). Notably, TNF-α has also been shown to directly promote FOXP3 degradation in Tregs (Gao et al. 2015), providing mechanistic insight into how inflammatory cytokines destabilize Treg identity and reinforcing the conclusion that TNF-α-mediated signals contribute to the loss of immunoregulatory balance during MASH.

Previous studies indicate that Forkhead box O (FOXO) transcription factors function as redox-sensors: oxidative stress can alter their post-translational modification, subcellular localization, and transcriptional activity, thereby modulating antioxidant defense and cellular metabolism (Essers et al. 2004; Klotz et al. 2015). Indeed, the dysregulation of FOXO activity contributes to hepatic steatosis, inflammation, and fibrogenesis by impairing lipid oxidation, promoting lipogenesis, and altering the inflammatory response. Specifically, loss of FOXO activity has been associated with enhanced hepatic triglyceride accumulation, pro-inflammatory cytokine production, and increased susceptibility to diet-induced steatohepatitis and fibrosis (Zhang et al. 2016). In the context, it is conceivable that lipid accumulation and associated oxidative stress in hepatocytes create a redox-imbalanced environment that may similarly impact FOXO activity in neighboring immune cells, including Tregs – leading to impaired FOXO–Treg axis function. Although direct experimental evidence for this pathway in MASLD remains limited, recent studies have shown that Foxo1 and Foxo3 directly bind to the promoter region of *Foxp3*, thereby promoting its transcription and ensuring sufficient Foxp3 protein levels in Tregs. Furthermore, FOXO factors regulate the expression of additional key molecules involved in Treg function, such as CTLA-4 and IL-10 (Hedrick et al. 2012; Williams and Chatila 2013). Taken together, the FOXO–Treg axis may serve as a key link between metabolic stress and immune dysregulation in MASLD, possibly through oxidative stress–mediated disruption of FOXO-dependent transcription in Tregs.

Krüppel-like factor 10 (KLF10) is a well-characterized transcription factor induced by TGF-β that plays a pivotal role in regulating the function and differentiation of both Teffs and Tregs (Cao et al. 2009). A recent study reported that KLF10 expression is significantly downregulated in Teffs and Tregs isolated from the peripheral blood and spleen of high-fat diet-induced obese mice (Wara et al. 2020). Moreover, the conditional deletion of *KLF10* in CD4^+^ T cells results in reduced Treg accumulation, leading to adipose tissue inflammation, insulin resistance, obesity, and the development of MASLD (Wara et al. 2020). Notably, the adoptive transfer of Tregs into CD4^+^ T cell-specific KLF10-deficient mice attenuates obesity, insulin resistance, adipose inflammation, and hepatic steatosis, underscoring the protective role of KLF10-expressing Tregs in metabolic liver diseases (Wara et al. 2020).

The nuclear receptor subfamily 4A (Nr4a) family, which consists of nuclear orphan receptor-type transcription factors, plays an essential role in the induction of Tregs within the thymus and maintenance of Treg populations in peripheral tissues (Sekiya et al. 2011; Sekiya et al. 2013). In a diet-induced MASH model, intrahepatic T cells showed increased *Nr4a* expression, and T cell–specific deletion of *Nr4a1* and *Nr4a2* resulted in attenuated MASH pathology accompanied by increased hepatic Treg accumulation (Aki et al. 2024; Hayatsu et al. 2017). Additionally, *Nr4a3* expression was upregulated, along with the increased expression of basic leucine zipper ATF-like transcription factor (Batf), a key regulator of Treg proliferation and function (Aki et al. 2024). Notably, Batf has been shown to be critical for Treg residency in non-lymphoid tissues (Hayatsu et al. 2017). Collectively, these findings suggest that Nr4a and Batf cooperatively promote the protective function of hepatic Tregs during liver inflammation, primarily by promoting their expansion and tissue retention.

In addition, lipid accumulation within hepatocytes contributes to the initiation of inflammation, which in turn promotes insulin resistance (IR) (Ipsen et al. 2018), a key driver in the progression of MASLD (Palma et al. 2022). IR disrupts the normal lipid metabolism by enhancing lipolysis and stimulating *de novo* lipogenesis, thereby sustaining hepatic fat accumulation (Petersen et al. 2007). This self-reinforcing cycle exacerbates liver inflammation, ultimately leading to hepatocyte injury and fibrotic progression (Powell et al. 2021). Visceral adipose tissue (VAT) Tregs, which express *PPARγ* and *ST2* (also known as the IL-33 receptor), have been reported to play a role in regulating systemic insulin sensitivity (Kolodin et al. 2015; Molofsky et al. 2015; Vasanthakumar et al. 2015). VAT-Tregs can lower fasting blood glucose levels and improve insulin resistance, thereby reducing inflammation and promoting metabolic health (Han et al. 2015; Winer et al. 2009). However, under conditions of obesity, these adipose Tregs are reduced in number and lose their suppressive capacity, which exacerbates adipose tissue inflammation and indirectly promotes hepatic steatosis and liver injury(Feuerer et al. 2009; Kawai et al. 2021). These observations highlight that not only hepatic Tregs but also extrahepatic Treg populations, particularly those in the VAT, are critical contributors to the immunometabolic crosstalk underlying MASLD progression.

## 6. The flip side: pathogenic roles of Tregs in liver fibrosis

Tregs in MASLD and MASH exhibit paradoxical functions: although they are well-recognized for their immunosuppressive properties, they may simultaneously contribute to the progression of MASH-associated liver fibrosis. Beyond their protective roles, Tregs engage in complex interactions with HSCs, the principal effector cells driving fibrogenesis in MASLD and MASH (Carter and Friedman 2022; Wang et al. 2022). While Tregs secrete anti-inflammatory cytokines like IL-10, IL-35, and TGF-β (Dikiy and Rudensky 2023), they can paradoxically promote HSC activation through TGF-β-dependent signaling pathway and IL-8 induction, thereby facilitating fibrogenesis (Dewidar et al. 2019; Langhans et al. 2013; Zhu et al. 2015). Notably, TGF-β, despite its immunoregulatory functions, is also a potent pro-fibrotic cytokine in liver disease; under conditions of sustained hepatic injury, TGF-β promotes HSC activation and their transdifferentiation into ECM-producing myofibroblasts, thus driving fibrogenic progression (Dewidar et al. 2019; Tsuchida and Friedman 2017).

The activating transcription factor 4 (ATF4), a basic leucine zipper transcription factor, is essential for cellular stress responses, particularly the integrated stress response (Pakos-Zebrucka et al. 2016). In conventional CD4^+^ T cells, ATF4 is involved in regulating metabolic reprogramming (Yang et al. 2018). A recent report further demonstrated that *Atf4* expression is enriched in hepatic Tregs from Western diet-induced obese mice, where it contributes to liver fibrosis by promoting TGF-β activation through integrin αvβ8. Therefore, blockade of integrin αvβ8 decreased active TGF-β levels in the livers of mice and attenuated fibrosis, underscoring the role of the ATF4–integrin αvβ8 axis in Treg-mediated fibrogenesis (Brown and Marshall 2019; Wang et al. 2025).

In both murine and human MASH livers, Tregs accumulate a distinct subset that produces amphiregulin (AREG), an epidermal growth factor receptor (EGFR) ligand. While AREG is known to facilitate tissue repair following acute injury in the context of chronic liver disease, Treg-derived AREG activates HSCs via EGFR signaling, thereby driving profibrotic transcriptional programs. Notably, Treg-specific deletion of *Areg*, but not deletion in myeloid cells, attenuated liver fibrosis in multiple diet-induced MASH models without significantly altering hepatic lipid accumulation or inflammation. Furthermore, the Treg-derived AREG promoted hepatocyte gluconeogenesis via HSC-derived IL-6, thereby linking liver fibrosis to systemic glucose intolerance. Importantly, mice lacking *Egfr* specifically on HSCs, were protected from both fibrosis and glucose dysregulation, highlighting the pathological significance of the Treg–AREG–HSC axis. Collectively, these findings challenge the traditional view that Tregs are solely protective in metabolic liver disease, and reveal their context-dependent roles in maladaptive tissue repair and metabolic regulation in MASH (Savage et al. 2024).

**Figure 2.**
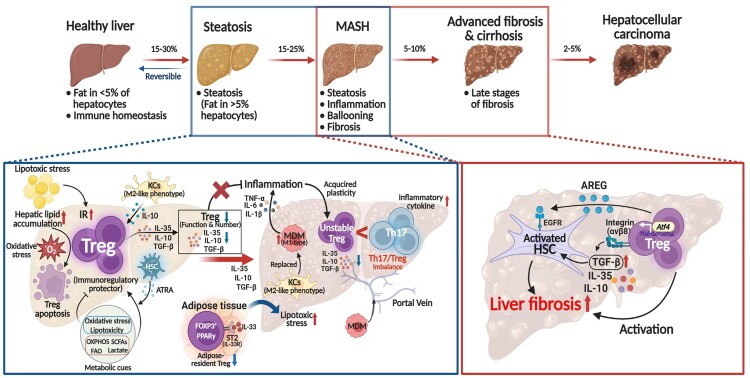
Stage-dependent functional transition of Tregs in MASLD progression. Tregs exhibit stage-dependent functional changes throughout the progression of MASLD, shifting from hepatoprotective roles in early disease to pro-fibrotic functions in later stages. In the healthy liver, Tregs contribute to immune tolerance and tissue homeostasis. During early steatosis, these hepatoprotective and immunoregulatory functions are maintained: HSCs produce ATRA, which promotes FOXP3^+^ Treg development even under inflammatory conditions; M2-like KCs secrete IL-10, supporting tissue repair and Treg differentiation; and metabolic cues such as OXPHOS, ATRA derived from HSCs, SCFAs, fatty FAO, and lactate contribute to Treg differentiation and lineage stability. However, oxidative stress and lipotoxicity begin to impair Treg survival and function during this phase. In the liver, excessive lipid accumulation and insulin resistance induce hepatocyte injury, leading to a pro-inflammatory and oxidative microenvironment that induces Treg apoptosis and promotes FOXP3 degradation. Consequently, Treg lineage stability is lost, ultimately resulting in a functional imbalance between regulatory T cells and pro-inflammatory Th17 cells, which exacerbates immune dysregulation during MASLD progression. Persistent hepatocellular damage and chronic inflammation result in KC depletion and infiltration of MDMs, which partially replace the lost KCs. These infiltrating MDMs exacerbate hepatic inflammation by producing pro-inflammatory cytokines including IL-1β, TNF-α, and IL-6. At the same time, adipose-derived IL-33 activates ST2^+^ PPARγ^+^ adipose-resident Tregs in VAT, promoting local immune regulation. As adipose tissue expands with worsening metabolic dysfunction, these VAT-Tregs decline due to increased inflammation and metabolic stress, which secondarily contributes to liver inflammation. As disease progresses, Tregs acquire a pro-fibrotic phenotype. Treg-derived TGF-β directly activates HSCs, promoting fibrogenesis. In addition, recent findings show that *ATF4* expression is elevated in hepatic Tregs during fibrosis, where it enhances TGF-β activation via integrin αvβ8. Moreover, Treg-derived AREG further stimulates HSCs through EGFR signaling. These combined signals perpetuate the activation of fibrogenic pathways and accelerate the progression toward irreversible cirrhosis. AREG; amphiregulin, ATF4; activating transcription factor 4, ATRA; all-trans retinoic acid, EGRF; epidermal growth factor receptor, FAO; fatty acid oxidation, HSCs; hepatic stellate cells, IR; insulin resistance, KCs; Kupffer cells, MDMs; monocyte-derived macrophages, OXPHOS; oxidative phosphorylation, PPARγ; peroxisome proliferator-activated receptor gamma, TGF-β; transforming growth factor-beta, TNF-α; tumor necrosis factor-alpha, SCFAs; short-chain fatty acids, VAT; visceral adipose tissue.

In summary, Tregs exhibit dual context-dependent functions in the pathogenesis of MASLD and MASH/advanced fibrosis (Figure 2). Although Tregs are classically recognized for their immunosuppressive and anti-inflammatory properties, emerging evidence has revealed a paradoxical role of Tregs in chronic liver disease. Under persistent inflammatory and metabolic stress, Tregs may undergo phenotypic and functional shifts that contribute to hepatic fibrosis and metabolic dysregulation. Therefore, a comprehensive understanding of Treg dynamics, tissue-specific interactions, and metabolic reprogramming is critical for the development of effective and safe Treg-targeted immunotherapies for MASLD and MASH.

## 7. Therapeutic targeting of Tregs: promise and pitfalls

Therapeutic strategies targeting Tregs in the context of MASLD and MASH have largely focused on enhancing their immunosuppressive function or expanding their population within affected tissues. Given the pivotal role of Tregs in suppressing chronic inflammation and preserving immune homeostasis, augmenting Treg-mediated regulation has emerged as a promising approach for mitigating liver injury and halting disease progression. Several preclinical studies have explored interventions that modulate Treg activity in experimental models of metabolic liver disease.

Several molecular and metabolic interventions have been used to enhance the intrinsic function of Tregs. For example, upregulation of miR-195, which suppresses CD40 expression, or direct CD40 silencing has been shown to correct the Th17/Treg imbalance, thereby attenuating hepatic inflammation and improving liver function in MASLD models (Li et al. 2020). Similarly, treatment with the antioxidant Mn(III)tetrakis (4-benzoic acid) porphyrin chloride protects hepatic Tregs from oxidative stress–induced apoptosis, leading to increased intrahepatic Treg abundance and reduced liver inflammation (Ma et al. 2007). In parallel, cellular approaches such as the adoptive transfer of ex vivo–expanded Tregs have been explored. Several studies have shown that adoptive Treg transfer can reduce intrahepatic TNF-α signaling, dampen hepatotoxic responses to LPS, and alleviate hepatic inflammation in MASLD models (Jin et al. 2025; Ma et al. 2007). CD73, an ectoenzyme critical for the immunosuppressive function of Tregs (Deaglio et al. 2007; Ohta and Sitkovsky 2014), has also been implicated, as its deficiency impairs Treg survival and exacerbates MASLD progression. Adoptively transferred WT Tregs, compared to CD73-knockout Tregs, enhanced intrahepatic Treg activity and reduced hepatic inflammation in MASLD (Jin et al. 2025).

Moreover, at the metabolic level, Tregs rely on basal mTORC1 activity driven by T cell antigen receptor and IL-2 signaling to program their suppressive function. This includes the regulation of cholesterol and lipid metabolism via the mevalonate pathway, which enhances the expression of key inhibitory molecules, such as CTLA-4 and ICOS (Zeng et al. 2013). Consequently, pharmacological agents that modulate mTOR signaling, including rapamycin, have emerged as promising candidates for restoring immune tolerance and reducing inflammation in chronic liver disease (Chapman and Chi 2014). In addition, peroxisome proliferator-activated receptors (PPARs), particularly PPARγ expressed in adipose tissue–resident Tregs, have emerged as important regulators of metabolic inflammation (Jakkawanpitak et al. 2026; Lee et al. 2025; Lefterova et al. 2014; Vasanthakumar et al. 2015). PPARγ agonists, such as the thiazolidinedione drug pioglitazone, exhibit anti-inflammatory properties and are considered promising therapeutic candidates for enhancing adipose tissue–Tregs function and restoring immune tolerance in chronic liver diseases (Negrotto et al. 2016; Tian et al. 2017).

However, despite these encouraging results, conflicting evidence exists regarding the safety and efficacy of Treg-based therapies, as adoptive Treg transfer has been reported to exacerbate MASH progression in certain dietary models. In the MASH-hepatocellular carcinoma (HCC) mouse model (stelic animal model), the depletion of Tregs markedly reduced steatosis and hepatocyte injury during the early disease stages, indicating a stage-specific pathogenic role for Tregs, where their depletion can attenuate liver damage (Wang et al. 2021b). Furthermore, in mice fed a high-fat and high-fructose diet, adoptive transfer of splenic Tregs significantly worsened hepatic steatosis (Van Herck et al. 2020). Similarly, adoptive Treg transfer and anti-CD3 antibody treatment are associated with increased hepatic steatosis and alanine aminotransferase levels under metabolic stress (Dywicki et al. 2022). Moreover, a reduction in Tregs is associated with the suppression of hepatocellular carcinoma development in NASH-associated liver cancer models. Human studies have similarly yielded conflicting results, with some studies reporting increased intrahepatic Treg numbers, whereas others have observed reduced frequencies in patients with MASLD (Bertola et al. 2010; Dywicki et al. 2022; Wang et al. 2021c).

These divergent observations underscore the complex, context-dependent roles of Tregs across disease stages (Table 1), particularly during the transition from MASH to HCC, where their immunosuppressive and tissue remodeling activities may shift from protective to pathogenic. Notably, recent evidence indicates that Tregs exert pro-fibrotic effects by promoting HSC activation, thereby exacerbating liver fibrosis. Therefore, therapeutic approaches targeting Tregs in MASLD and MASH should be carefully tailored to the disease stage, recognizing that strategies aimed at enhancing Treg function in the early inflammatory phases may be beneficial, whereas selective modulation or suppression of Tregs might be warranted in advanced fibrosis or HCC settings to prevent excessive tissue remodeling and restore effective antitumor immunity.

**Table 1.** Liver-Treg function in MASLD progression.

Disease stage	Function of Treg	Outcome	References
Early Steatosis	Treg maintain immune homeostasis by restraining Th1/Th17-mediated inflammation and preventing initial hepatocyte injury.	Disease progression is accelerated when Tregs are reduced via apoptosis or instability.	(Ma et al. 2007) (Tang et al. 2011) (Winer et al. 2009)
Steatohepatitis (MASH)	Chronic inflammatory signals destabilize FOXP3 and decrease Treg abundance, compromising their suppressive activity.	Inflammation worsens, promoting transition from steatosis to steatohepatitis.	(Roh et al. 2018) (Sutti and Albano 2020) (Li et al. 2021)
Fibrosis	Tregs acquire pro-fibrotic properties through: TGF-β–dependent HSC activation ATF4–integrin αvβ8 pathway Areg–EGFR signaling in HSCs	Paradoxically, Tregs promote fibrosis and metabolic dysregulation.	(Wang et al. 2025) (Savage et al. 2024) (Langhans et al. 2013) (Aki et al. 2024)
MASH-HCC Transition	High Treg activity may favor immune evasion and tumor tolerance	Disease progression accelerates toward HCC development.	(Wang et al. 2024a, 2024b) (Wang et al. 2021a, 2021b) (Ma et al. 2016)

## 8. Future perspective and conclusion remarks

Although the recent FDA approval of resmetirom (Rezdiffra) for MASH represents an important step forward in the clinical management of metabolic liver disease (Brisnovali et al. 2024; Hasan et al. 2025), the complexity of MASLD pathogenesis, particularly its immunometabolic dimensions, calls for the development of broader, mechanism-based therapeutic strategies. A promising area of investigation is the modulation of Tregs, which have emerged as critical players in the hepatic immune landscape.

Tregs play an essential role in maintaining hepatic immune tolerance and suppressing excessive inflammation. However, accumulating evidence suggests that Tregs are highly plastic and metabolically responsive, and that their roles may vary depending on the disease stage and microenvironmental cues. In the early stages of disease, Tregs may act as protective agents by limiting inflammation and injury. However, under chronic stimulation or altered phenotypic states, they may inadvertently contribute to fibrogenesis by modulating HSC activation or promoting an immunosuppressive milieu that facilitates HCC progression.

As the field progresses, a deeper understanding of the molecular mechanisms regulating Treg plasticity, trafficking, and effector function in the steatotic liver is needed. Emerging tools such as single-cell transcriptomics, spatial omics, and metabolic profiling of immune cells will be invaluable for defining context-specific Treg subsets and their interactions with metabolic and stromal components in MASLD. Furthermore, targeting Treg pathways, whether through cytokine signaling modulation or metabolite-based interventions, holds promise as a future therapeutic avenue. Importantly, MASLD is closely associated with systemic metabolic dysfunction, including insulin resistance, adipose tissue inflammation, and altered lipid handling. Therefore, Treg-targeted therapies must also consider the potential extrahepatic effects to avoid unintended consequences on whole-body metabolic homeostasis. Despite encouraging advances in preclinical models, clinical translation of Treg-based therapies remains limited, highlighting the need for rigorous experimental validation and biomarker-guided approaches to ensure both efficacy and safety.

In conclusion, Tregs play a central role at the intersection of immunity and metabolism in patients with MASLD and MASH. A more refined understanding of their regulatory networks is essential to unlock their therapeutic potential and complement existing and emerging metabolism-focused treatments for this increasingly prevalent disease.
